# Knowledge of Health Effects and Intentions to Quit Among Smokers in India: Findings From the Tobacco Control Policy (TCP) India Pilot Survey

**DOI:** 10.3390/ijerph9020564

**Published:** 2012-02-15

**Authors:** Genevieve C. Sansone, Lalit J. Raute, Geoffrey T. Fong, Mangesh S. Pednekar, Anne C. K. Quah, Maansi Bansal-Travers, Prakash C. Gupta, Dhirendra N. Sinha

**Affiliations:** 1 Department of Psychology, University of Waterloo, 200 University Avenue West, Waterloo, Ontario N2L3G1, Canada; Email: gfong@uwaterloo.ca (G.T.F.); ackquah@uwaterloo.ca (A.C.K.Q.); 2 Healis Sekhsaria Institute for Public Health, 601/B, Great Eastern Chambers, Plot No. 28, Sector 11, CBD Belapur, 400 614, Navi Mumbai, India; Email: rautel@healis.org (L.J.R.); pednekarm@healis.org (M.S.P.); pcgupta@healis.org (P.C.G.); 3 Ontario Institute for Cancer Research, MaRS Centre, South Tower, 101 College Street, Suite 800, Toronto, Ontario M5G 0A3, Canada; 4 Roswell Park Cancer Institute, Elm and Carlton Streets, Buffalo, NY 14263, USA; Email: Maansi.Travers@roswellpark.org; 5 School of Preventive Oncology, A/27, Anandpuri, West Boring Canal Road, 800001, Patna, Bihar, India; Email: dhirendrasinha1@gmail.com

**Keywords:** health risks, smoking, health knowledge, quit intentions, India

## Abstract

Awareness of the health risks of smoking is an important factor in predicting smoking-related behaviour; however, little is known about the knowledge of health risks in low-income countries such as India. The present study examined beliefs about the harms of smoking and the impact of health knowledge on intentions to quit among a sample of 249 current smokers in both urban and rural areas in two states (Maharashtra and Bihar) from the 2006 TCP India Pilot Survey, conducted by the ITC Project. The overall awareness among smokers in India of the specific health risks of smoking was very low compared to other ITC countries, and only 10% of respondents reported that they had plans to quit in the next six months. In addition, smokers with higher knowledge were significantly more likely to have plans to quit smoking. For example, 26.2% of respondents who believed that smoking cause CHD and only 5.5% who did not believe that smoking causes CHD had intentions to quit (*χ*^2^ = 16.348, *p* < 0.001). Important differences were also found according to socioeconomic factors and state: higher levels of knowledge were found in Maharashtra than in Bihar, in urban compared to rural areas, among males, and among smokers with higher education. These findings highlight the need to increase awareness about the health risks of smoking in India, particularly in rural areas, where levels of education and health knowledge are lower.

## 1. Introduction

Almost one million annual deaths from tobacco-related diseases occur in India, the world’s second-largest consumer of tobacco, where about one-third of adults use some form of tobacco [1,2,3]. India has a long history of tobacco use, of which cigarette smoking is only a minor part; according to the Global Adult Tobacco Survey [1], the majority of tobacco users (60%) consume only smokeless tobacco and even among smokers, bidis (which are made by rolling tobacco in a tendu leaf) are much more commonly smoked than cigarettes. Although India ratified the World Health Organization’s Framework Convention on Tobacco Control (FCTC) on February 2004, the government continues to struggle with effective implementation of the important policy changes required by the treaty.

Besides these policy barriers, another challenge for effective tobacco control in India is education and awareness among the population. Knowledge of the health effects of smoking is an important factor in predicting smoking-related behaviour, including lower likelihood of initiation and greater likelihood of quitting [4,5,6,7]. However, the majority of research on awareness of harms has been conducted among cigarette smokers in Western countries; much less is known about the awareness of harms of tobacco use among users of other forms of tobacco in developing countries such as India.

It is also important to consider the role of socioeconomic factors in levels of knowledge and awareness in low- and middle-income countries. There is clear evidence that tobacco use and its health effects are associated with poverty and illiteracy, both at the individual and the country level [8,9]. Research from Western countries suggests that knowledge of the health effects of tobacco tends to be lower among population groups with lower socioeconomic status [6,10,11].

India is categorized as a lower-middle-income country, with about 37% of the population estimated to live below the poverty line [12]. The rate of illiteracy is also high compared to other countries, with 39% considered illiterate [7]. In addition, as per the 2011 Census, the majority of the population (69%) lives in rural areas, where people tend to have less education [13]. Studies in India have shown higher rates of smoking prevalence among the illiterate population [1,8,14]. A recent study also found lower knowledge of the health effects of smokeless tobacco among smokeless users in rural areas in the state of Maharashtra compared to those in urban areas [15]. These factors may therefore contribute to lower overall levels of awareness of the health risks of tobacco in India, particularly among those with lower education and income.

The aim of the present study was to assess beliefs about the harms of smoking and the relation between health knowledge and intentions to quit among a sample of 249 current smokers across two states in India (Maharashtra and Bihar). A secondary aim was to examine differences in knowledge across different areas of India, as well as differences according to socioeconomic factors.

## 2. Methods

### 2.1. Data Source

The Tobacco Control Project (TCP) India Pilot Study is a cross-sectional survey conducted in 2006 as a lead-in to the TCP India Project, a larger prospective cohort study which began in 2010. The TCP India Pilot Study, as with the TCP India Project and all other International Tobacco Control (ITC) Policy Evaluation Projects being conducted in more than 20 countries, was designed to evaluate and understand the psychosocial and behavioural effects of national-level tobacco control policies [16,17,18]. In India, the International Tobacco Control Policy Evaluation Project (ITC Project) was renamed as the “Tobacco Control Policy (TCP) India Pilot Study Survey” to avoid confusion with the “India Tobacco Company.”

### 2.2. Sampling Design and Procedures

The TCP India Pilot Study was conducted in four areas of two states: the urban areas of Mumbai and Patna, and their surrounding rural areas in the states of Maharashtra and Bihar respectively. The target population included an approximately equal distribution of adult smokers, smokeless tobacco users and non-users of tobacco aged 18 and older.

Data for the survey were collected through face-to-face interviewing techniques in households that were randomly selected through probability sampling methods. The survey was conducted in either Hindi (in Bihar) or Marathi (in Maharashtra) and respondents were given a small token of appreciation at the end of the session. Additional information about the research design and survey methodologies has been reported in Raute *et al.* [15] and is available at the ITC Project website: www.itcproject.org. The study protocol and survey materials were approved by the Office of Research Ethics at the University of Waterloo, Canada and the Healis-Institutional Review Board at the Healis-Sekhsaria Institute for Public Health, India.

### 2.3. Measures

The TCP India Pilot Survey was standardized with ITC Surveys conducted in other countries so that respondents are asked the same questions in each country, with only minor variations to accommodate cultural or language differences. The TCP India Pilot Survey included questions about self-reported smoking behavior, policy-relevant variables, and psychosocial measures such as knowledge and attitudes towards smoking, as well as intentions to quit. 

#### 2.3.1. Demographics

The following demographic measures were included in the analyses: sex, age, education, and income. Age was divided into four categories: 18–24 years, 25–39 years, 40–54 years, and 55 and older. Respondents’ highest level of education was measured according to six categories. In order to standardize these categories, we divided respondents into three groups: low (illiterate or primary), moderate (middle or secondary), or high (college or above) education. Household income levels were also categorized as low (<5,000 INR per month), middle (5,000–15,000 INR per month) or high (>15,000 INR per month). We also created an SES variable as a function of the standardized education and income levels. However, this combined variable did not provide any significant information beyond the contribution of education, so we report only on the individual education and income variables in the analyses, and the SES variable is not discussed further in this paper.

#### 2.3.2. Variables of Interest

All respondents were asked a series of questions regarding their knowledge and beliefs about different tobacco products, including smoked and smokeless tobacco. Current smokers were asked about their opinion of smoking as well as their beliefs about the harm caused by smoking and more specifically, whether smoking has damaged their own health. Knowledge of the specific health effects of smoking was assessed by asking smokers whether they believed smoking causes the following health outcomes: stroke, coronary heart disease, impotence, lung cancer, mouth cancer, stained teeth, premature ageing in smokers, and lung cancer in non-smokers. Responses were coded as 0 = “no/don’t know” *vs.* 1 = “yes”. A health knowledge scale was then created by summing the number of “yes” responses across the 8 diseases/health effects to create a single score. However, because the mouth cancer question was missing in Maharashtra, this question was not included in the knowledge scale so the range of scores on the scale was from 0 to 7.

Finally, intentions to quit among smokers were measured with the question “Are you planning to quit smoking…within the next month; within the next 6 months; sometime in the future, beyond 6 months; or not planning to quit”. A dichotomous variable was then created so that responses of “within the next month/6 months” = having intentions to quit, and responses of “beyond 6 months/not at all” = no intention.

### 2.4. Data Analysis

SPSS Version 16 was used for all statistical analyses. Analyses were performed only on the data from current smokers (N = 249) in the overall sample of 764 respondents. A smoker was defined as someone who smokes cigarettes or bidis at least weekly. Chi-square tests were conducted to examine bivariate differences in all the measures between the two states. For the knowledge questions, we examined knowledge of each health effect separately, as well as using the combined health knowledge scale described in the previous section. We further examined correlations between scores on the knowledge scale with education and income levels. We also conducted linear regression and separate multivariate logistic regression analyses to examine factors that might predict scores on the knowledge scale and the odds of knowing that smoking causes each of the specific health effects. Several demographic factors were included in the analyses, including sex, age group, marital status, religion, state, urban/rural area, income level and education level. Finally, the association between knowledge of each of the health effects and intentions to quit was examined using chi-square analyses.

## 3. Results

### 3.1. Characteristics of the Sample

The socio-demographic characteristics for the sample of smokers are presented in Table 1. Full details of the complete sample are available in Raute *et al.* [15]. Approximately equal numbers of smokers were sampled in each state, with a higher proportion sampled in the rural areas within each state compared to the urban area, particularly in Bihar, where 83% of the sample was rural. Overall, the majority of the smokers sampled were male. The proportion of males was much higher in Maharashtra (93.9%) than in Bihar (57.8%). The sample was roughly equally distributed across the three older age groups, with a low proportion of 18–24 year olds in all areas. Overall, 60.9% of the sample was classified as having a low education level, and 74.1% had a low income level. For both of these variables, the proportion of the sample in the lowest category was much higher in rural areas than in urban areas, for both states. There was also a higher proportion of low education respondents in Bihar (64.2%) compared to Maharashtra (57.0%).

**Table 1:** Sample characteristics by state and urban/rural area.

Variables	Maharashtra			Bihar			Overall N (%)
	Urban N (%)	Rural N (%)	Total N (%)	Urban N (%)	Rural N (%)	Total N (%)	
	46 (40.4)	68 (59.6)	114 (45.8)	23 (17.0)	112 (83.0)	135 (54.2)	249 (100.0)
Sex							
Male	44 (95.7)	63 (92.6)	107 (93.9)	18 (78.3)	60 (53.6)	78 (57.8)	185 (74.3)
Female	2 (4.3)	5 (7.4)	7 (6.1)	5 (21.7)	52 (46.4)	57 (42.2)	64 (25.7)
Age group							
18–24	4 (8.7)	2 (2.9)	6 (5.3)	2 (8.7)	5 (4.5)	7 (5.2)	13 (5.2)
25–39	14 (30.4)	9 (13.2)	23 (20.2)	2 (8.7)	39 (34.8)	41 (30.4)	64 (25.7)
40–54	11 (23.9)	36 (52.9)	47 (41.2)	7 (30.4)	34 (30.4)	41 (30.4)	88 (35.3)
55+	17 (37.0)	21 (30.9)	38 (33.3)	12 (52.5)	34 (30.4)	46 (34.1)	84 (33.7)
Education level							
Low	11 (23.9)	54 (79.4)	65 (57.0)	6 (26.1)	80 (72.1)	86 (64.2)	151 (60.9)
Middle	28 (60.9)	12 (17.6)	40 (35.1)	11 (47.8)	21 (18.9)	32 (23.9)	72 (29.0)
High	17 (37.0)	2 (2.9)	9 (7.9)	6 (26.1)	10 (9.0)	16 (11.9)	25 (10.1)
Income level							
Low	21 (46.7)	61 (89.7)	82 (72.6)	7 (33.3)	90 (81.8)	97 (74.0)	179 (73.4)
Middle	14 (31.1)	7 (10.3)	21 (18.6)	9 (42.9)	16 (14.5)	25 (19.1)	46 (18.9)
High	10 (22.2)	0 (0.0)	10 (8.8)	5 (23.8)	4 (3.6)	9 (6.9)	19 (7.8)

### 3.2. Smoking-Related Beliefs and Intentions to Quit

Table 2 presents the responses for our variables of interest and the tests of significance for differences in responses between the two states. Overall, smokers had a negative opinion of smoking and believed that smoking is bad for their health. The majority of smokers (79%) agreed that smoking is ‘not good’ for their health, with more smokers in Bihar believing smoking is not good for health than in Maharashtra (86% *vs.* 72%). In addition, 70% of smokers overall had a bad or very bad opinion of smoking, which did not differ between the two states.

**Table 2:** Variables associated with knowledge and attitudes towards smoking by state.

Variable	Maharashtra (N = 114)		Bihar (N = 135)		Overall (N = 249)	
	N	%	N	%	N	%
Think smoking is good for health *						
good	22	19.5	14	10.4	36	14.6
neither good nor bad	10	8.8	5	3.7	15	6.1
not good	81	71.7	115	85.8	196	79.4
Overall opinion about smoking						
good or very good	22	19.3	14	10.5	36	14.6
neither good nor bad	13	11.4	24	18.0	37	15.0
bad or very bad	79	69.3	95	71.4	174	70.4
In the last month, how often you thought about the harm your smoking might be doing to you ***						
never	64	56.6	55	45.5	119	50.9
sometimes	28	24.8	89	50.4	89	38.0
often	21	18.6	26	4.1	26	11.1
Extent smoking has damaged health ***						
not at all	76	67.3	34	25.2	110	44.4
a little	31	27.4	59	43.7	90	36.3
very much	6	5.3	1	0.7	7	2.8
don’t know/cannot say	0	0.0	41	30.4	41	16.5
Rating of overall health ^α^	3.98		4.10		4.04	
In the last month, how often you seriously considered quitting smoking *						
Never	68	60.2	70	56.9	138	58.5
Sometimes	33	29.2	50	40.7	83	35.2
Often	12	10.6	3	2.4	15	6.4
Intention to quit smoking **						
Within the next month	9	8.6	1	1.0	10	4.9
Within the next 6 months	7	6.7	3	3.0	10	4.9
Sometime in the future, beyond 6 months	20	19.0	37	36.6	57	27.7
Not planning to quit	69	65.7	60	59.4	129	62.6

^α^ 1 = poor, 5 = excellent. * p < 0.05; ** p < 0.01; *** p < 0.001.

However, the majority of smokers also believed that they are in good health and did not perceive their smoking habit to be harming them. About half of the smokers in the sample (51%) reported that they never thought about the harm their smoking might be doing to them in the past month, and this proportion was higher in Maharashtra than in Bihar (57% *vs.* 46%). In addition, 44% overall believed that smoking has not damaged their health at all. This proportion was significantly higher in Maharashtra (67%) than in Bihar (25%), *χ*^2^ = 43.12, *p* < 0.001. When asked to rate their overall health on a scale of 1 (poor) to 5 (excellent), smokers rated their health highly, with a mean rating of 4.04, which did not differ between the two states.

Overall, very few smokers had intentions to quit smoking. Only 10% of smokers reported that they intended to quit either within the next month or next 6 months and 37% had any intention to quit. Quit intentions were higher in Maharashtra (15.2%) than in Bihar (4.0%), *χ*^2^ = 7.47, *p* = 0.008. In addition, 59% of smokers reported that they never seriously considered quitting in the last month, and more smokers said they often considered quitting in Maharashtra (11%) than in Bihar (2%), *χ*^2^ = 6.62, *p* = 0.01.

### 3.3. Knowledge of Health Effects

**Figure 1:**
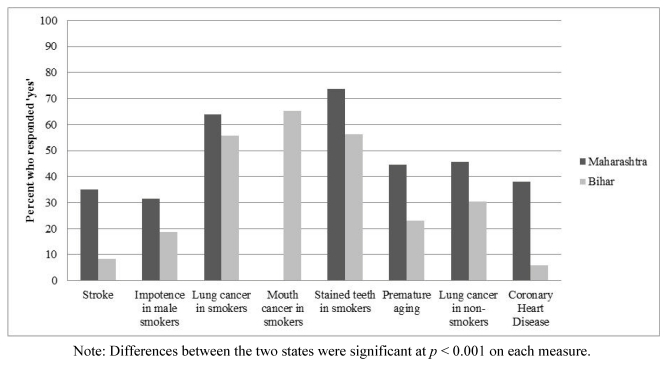
Knowledge of specific health effects by state.

Figure 1 presents the proportion of smokers in each state who believed that smoking causes each of the eight health effects. Overall knowledge levels across the two states were lowest for stroke (20.6%) and coronary heart disease (20.7%), and highest for stained teeth (64.3%) and mouth cancer in smokers (65.2%). However, the mouth cancer question was missing in Maharashtra, so the estimate for that question is based only on the data from smokers in Bihar. A greater proportion of smokers in Maharashtra believed that smoking causes each of the health effects for all measures, compared to Bihar. 

### 3.4. Logistic Regression Analyses

Of the demographic factors that were included in the multivariate logistic regression analyses, only four of these factors were significant predictors on any of the knowledge questions: state, urban/rural area, sex, and education level. Table 3 presents the odds ratios of knowing that smoking causes each of the health effects for each of these predictors, as well as the interaction between state and urban/rural area.

**Table 3:** Logistic Regression showing significant predictors (excludes age group, marital status, religion, and income level).

Adjusted odds ratios^†^ from logistic regression of knowledge of specific health effects (N = 249)										
Covariate		OR (95% CI)								*t* (95% CI)
		Stroke	Impotence	Lung Cancer in Smokers	Mouth Cancer	Stained Teeth	Premature Aging	Lung Cancer in Nonsmokers	Coronary Heart Disease	Knowledge Scale
**State**	MH	5.12 (2.02–12.95) **	1.55 (0.75–3.21)	1.12 (0.51–2.45)	n/a	1.86 (0.89–3.90)	2.03 (0.97–4.27)	1.45 (0.67–3.14)	15.40 (4.67–5.81) ***	−2.97 (−1.16–(−)0.24) **
	Bihar	Ref	Ref	Ref	Ref	Ref	Ref	Ref	Ref	
**Area**	Urban	3.74 (1.48–9.47) **	1.73 (0.75–3.99)	5.29 (1.65–16.96) **	2.49 (0.37–16.61)	4.63 (1.51–14.23) **	7.35 (3.08–17.54) ***	3.99 (1.64–9.71) **	9.76 (3.23–29.51) ***	−6.52 (−2.45–(−)1.31) ***
	Rural	Ref	Ref	Ref	Ref	Ref	Ref	Ref	Ref	
**Sex**	Male	3.33 (0.60–18.32)	1.88 (0.67–5.33)	2.20 (0.91–5.33)	2.12 (0.82–5.49)	1.17 (0.52–2.64)	3.07 (1.01–9.27) *	3.08 (1.00–9.46) *	1.17 (0.18–7.69)	−2.11 (−1.14–(−)0.04) *
	Female	Ref	Ref	Ref	Ref	Ref	Ref	Ref	Ref	
**Education Level**	Low	0.15 (0.04–0.61) **	0.31 (0.09–1.01)	0.11 (0.02–0.67) *	0.11 (0.01–1.01)	0.25 (0.05–1.19)	0.24 (0.07–0.83) *	0.05 (0.01–0.22) ***	0.15 (0.03–0.84) *	5.41 (0.64–1.37) ***
	Moderate	0.60 (0.18–1.99)	0.90 (0.31–2.60)	1.09 (0.17–7.06)	1.32 (0.11–17.52)	0.72 (1.51–3.46)	0.30 (0.09–0.94) *	0.26 (0.–.16) *	1.07 (0.24–4.68)	
	High	Ref	Ref	Ref	Ref	Ref	Ref	Ref	Ref	
**State x Area Interaction**		3.23 (0.50–20.73)	0.71 (0.16–3.19)	21.57 (1.52–306.67) *	n/a	9.69 (0.80–117.63)	2.41 (0.52–11.24)	3.54 (0.68–18.47)	5.74 (0.61–54.01)	3.36 (0.69–2.63) **

Significant levels are indicated as follows: *p < 0.05 **p < 0.01; ***p < 0.001. ^† ^Odds of responding that smoking causes each health effect (0: no/don’t know, 1: yes). MH = Maharashtra.

Knowledge about two of the health effects (stroke and coronary heart disease) was significantly higher in Maharashtra than in Bihar, and the odds ratios were also higher in Maharashtra for each of the other health effects, although not significantly higher. Odds were higher in urban areas for each of the eight health effects, although two of the health effects were not significant (impotence and mouth cancer). Males had consistently higher odds ratios than females (and significantly higher for premature aging and lung cancer in non-smokers). Smokers with low education had consistently lower odds than those with high education (although this was not significant for impotence, mouth cancer and stained teeth), and for two health effects (premature aging and lung cancer in non-smokers), those with moderate education also had significantly lower odds. Finally, the state by area interaction was only significant for one of the health effects: the urban-rural difference in knowledge of lung cancer in smokers was greater in Maharashtra than in Bihar.

### 3.5. Knowledge Scale Analyses

**Figure 2:**
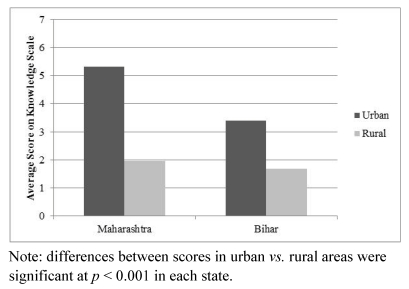
Scores on knowledge scale by state and urban/rural area.

Table 4 presents the scores on the knowledge scale by state and urban or rural area. The overall score for the sample was low (2.59). The mean score in Maharashtra was higher than in Bihar (3.32 *vs.* 1.97, *t* = 4.99, *p* < 0.001), and much higher in urban than in rural areas (4.68 *vs.* 1.79, *t* = 11.20, *p* < 0.001). This difference between urban and rural areas was also found to be significant in each state separately (see Figure 2). The highest knowledge scores were found in urban Maharashtra (Mumbai), where the mean score on the scale was 5.32.

**Table 4:** Scores on knowledge scale.

		Mean Score (N)
State ***	Bihar	1.97 (135)
	Maharashtra	3.32 (114)
Urban/Rural ***	Urban	4.68 (69)
	Rural	1.79 (180)
Overall		2.59 (249)

*** *p* < 0.001.

The same predictors from the multivariate logistic regression were then used to predict scores on the knowledge scale in a separate linear regression analysis (see Table 3). Consistent with the previous results, knowledge scores were significantly higher in Maharashtra, in urban areas of both states, among males and among those with higher education. The interaction between state and urban/rural area was also significant, due to the greater difference in knowledge scores between the urban and rural areas in Maharashtra compared to Bihar.

In addition, scores on this knowledge scale were found to be positively correlated with education level (*r* = 0.515, *p* < 0.001; *r* = 0.549 in Maharashtra and *r* = 0.547 in Bihar) and also with income level (*r* = 0.337, *p* < 0.001; *r* = 0.378 in Maharashtra and *r* = 0.312 in Bihar).

### 3.6. Relation Between Knowledge and Intentions to Quit

Smokers who believed that smoking causes each of the health effects were significantly more likely to intend to quit smoking than those who responded “no” or “don’t know”. This pattern was observed for each of the health effects, although it was not significant for two of the health effects: mouth cancer and impotence. For example, 26.2% of respondents who believed that smoking cause CHD and only 5.5% who did not believe that smoking causes CHD had intentions to quit (*χ*^2^ = 16.348, *p* < 0.001). There was also a significant effect for the overall score on the knowledge scale—smokers who intended to quit had a higher mean score than smokers who did not intend to quit (4.65 *vs.* 2.49, *t* = 4.45, *p* < 0.001).

## 4. Discussion

The results of this study showed that the overall awareness among smokers in India of the specific health risks of smoking was very low, compared to other countries. Research in developed countries has shown that the majority of smokers are aware of the major health effects of smoking, including lung cancer and stroke [6,19]. For instance, in a survey of smokers in four Western countries (USA, Canada, Australia and U.K.) conducted by the ITC Project, 94% believed that smoking causes lung cancer and 89% believed smoking causes heart disease [20].

However, this same level of knowledge has not been found in low- and middle-income countries, where levels of education and income are generally lower. In the 2010 ITC Bangladesh Survey, levels of knowledge of various health effects were lower than in Western countries, but still higher than the current levels found in the TCP India Pilot Study. For example, 85% of smokers in Bangladesh believed smoking causes lung cancer and 79% believed smoking causes heart disease [21]. Similar results were found in the 2009 ITC Bhutan Survey, where 86% of tobacco users believed that smoking causes lung cancer [22]. The results of the present study are more consistent with low knowledge levels found among Chinese smokers in a 2006 study using ITC China Survey data [23]. In this study, 68% of current smokers believed that smoking causes lung cancer, and only 16% believed smoking causes CHD, compared to 60% and 21% respectively in India. These results support global research indicating that despite the evidence for the harms caused by tobacco, the majority of tobacco users worldwide are not fully aware of the risks, other than lung cancer [7].

In addition, while the majority of smokers in this study had a negative opinion of smoking, most smokers overall were not concerned about the negative consequences of smoking on their own personal health. They maintain the perception that they are in good health, and they believe that smoking has not damaged their health.

Furthermore, only 10% of current smokers in this sample in India reported that they had plans to quit smoking in the next six months, and 37% had any intention to quit. This finding is in line with quit intentions in Bangladesh (10% of cigarette smokers in 2009 planned to quit in the next 6 months) [24] and in China (24% of smokers across six states in 2006 had any intention to quit) [25], but it is still much lower than rates of quit intentions in other countries. For instance, ITC Surveys in four Western countries have found rates of quit intentions of about 36% of smokers overall, with 65–81% having any intention to quit at some point in the future [20,26]. The level of quit intentions in this sample is also slightly lower than the level reported in the Global Adult Tobacco Survey (GATS 2010) in India, which found that 12% of current smokers planned to quit in the next month, and 26% in total planned to quit within the next year [1]. 

An important finding in this study was the relation between knowledge of the harms of smoking and intentions to quit. Respondents who were aware of the specific health effects of smoking were consistently more likely to have plans to quit smoking. Research has shown that quit intentions are a significant predictor of making an actual quit attempt [27]. Therefore, efforts to improve levels of awareness about the harms of smoking in India may be an important strategy for motivating smokers to quit and increasing successful quit attempts.

### 4.1. State Differences

There were significant differences in many of the results between smokers in these two states. Analyses of the differences between the states revealed higher levels of knowledge of the health risks, as well as stronger intentions to quit smoking in Maharashtra than in Bihar. However, the results also showed lower concern about the harms of smoking in Maharashtra on certain measures. For example, a higher proportion of smokers in Maharashtra reported that smoking has not damaged their health and that they never think about the harm their smoking does to them. They were also less likely to say that smoking is “not good” for health. 

The reasons for these observed differences across states are not clear. However, they imply that there may be a greater need for education and information programs about the harms of smoking in Bihar than in Maharashtra. The differences among smokers in various states in India will be explored further with the data from the full Wave 1 TCP India Survey, which is being conducted in four states: Bihar, Madhya Pradesh, Maharashtra and West Bengal.

### 4.2. Role of Socioeconomic Factors

Important differences in the results were also found according to socioeconomic factors and urban or rural area. Smokers in urban areas of both states had much higher levels of knowledge of the health risks of smoking compared to those in rural areas. Education level was also a significant predictor of knowledge – smokers in the lowest education category were consistently less likely to be aware of the harms of smoking. Moreover, education level was a stronger predictor than income level. However, these findings are based on a small sample that consisted of a high proportion of respondents with low education and income levels, particularly in the rural areas of Maharashtra and Bihar. 

### 4.3. Limitations of the Current Study

The present sample was limited to two states in India; therefore, these results are not representative of smokers in the country as a whole. The unequal distribution of the sample across education and income categories is another limitation of this study. Furthermore, the measure of income that was used was a measure of household income, not individual income, and it does not take into account the number of family members in the household that must be provided for. Therefore, we might expect greater variation in the impact of income in India because of the large variation in household sizes in the country. The expanded Wave 1 TCP India Survey will also provide more detail about actual income levels, rather than the basic categorical information that was available from the pilot study data.

Another limitation of the current study was the small sample size of respondents, which reduced the statistical power of the significance tests conducted. It should be noted, however, that the smaller sample size cannot be an explanation for the many comparisons that were found to be statistically significant, such as differences in knowledge and in quit intentions between the states and between urban and rural areas. These effects will be explored more thoroughly in the forthcoming TCP India Survey.

In addition, because of cross-sectional design of the study, we were only able to explore associations between our measures without addressing the causal relationship between health knowledge and our other variables. These associations will be addressed more thoroughly in the TCP India Survey, which employs a longitudinal cohort design.

### 4.4. Implications

The findings from this study highlight the need to increase awareness about the health effects of smoking in India to encourage quitting, particularly in rural areas, where levels of education and health knowledge are lower and where health care services are less available. One important method of increasing awareness is through warning labels [20,28]. Current pictorial warnings in India have been shown to be weak and ineffective, as they are poorly understood by most smokers [29,30].

Policymakers should also take into account the greater prevalence of smoking and lower knowledge of the health risks among lower SES groups. For example, higher taxes are an effective method of reducing consumption among smokers with lower SES, who are more price-sensitive [7,31]. However, it is important to note that lack of knowledge is not the only factor influencing the use of tobacco among socioeconomically disadvantaged groups, hence a comprehensive strategy that addresses environmental factors would be most successful [6].
